# Bridging the Gap in Studies on the Food Environment: The State-of-the-Art in LMICs

**DOI:** 10.3390/ijerph22121865

**Published:** 2025-12-15

**Authors:** Mariana Carvalho de Menezes, Ariene Silva do Carmo, Larissa Loures Mendes

**Affiliations:** 1 Department of Nutrition, Federal University of Minas Gerais, Belo Horizonte 31270-901, MG, Brazil; larissaloures@ufmg.br; 2 Department of Clinical and Social Nutrition, Federal University of Ouro Preto, Ouro Preto 35400-000, MG, Brazil; arienecarmo@gmail.com

Food systems influence population health through multiple pathways and are recognized as key drivers of the global syndemic of undernutrition, obesity, and climate change (Swinburn et al., 2019) [1]. A critical component of these systems is the food environment, defined as the set of physical, economic, political, and socio-cultural conditions that shape how people access, choose, and consume food (HLPE, 2017) [2]. By understanding the dynamics of food environments, it becomes possible to design and implement evidence-informed policies capable of reshaping them, thereby promoting healthier diets and addressing malnutrition in all its forms.

Despite rapid transformations in low- and middle-income countries (LMICs), much of the empirical literature on food environments has historically centered on high-income settings. LMICs require distinct approaches that reflect cultural diversity, structural inequities, informal markets, rapid urbanization, and the dual burden of malnutrition. This Special Issue, ‘’Bridging the Gap in Studies on the Food Environment: The State-of-the-Art in LMICs’’, answers that call by assembling together eight articles that address these complexities, ranging from conceptual modeling to spatial analyses and the decision matrix tool, together offering a more nuanced and context-sensitive portrait of food environments in LMICs.

**Evidence Across Settings: From Perception to Place**

**Avelar et al. (contribution 1)** used a mixed-methods approach to explore how patients from Primary Health Care perceived the local food environment. The study identified both barriers (such as the poor availability and high price of fresh food, easy access to ultra-processed foods strongly influenced by advertising, and transformations in food systems, such as making the most of food purchases at large supermarket chains and use of delivery services) and enablers of healthy eating (such as home gardens, food from family farming, street markets and the variety of fresh food close to home) offering grounded insights for community-based policies. These grounded insights underscore opportunities for community-based policies that build on existing assets while countering the structural drivers of unhealthy diets.

**De Souza et al. (contribution 2)** conducted a spatiotemporal analysis in a Brazilian city revealing a 63% increase in food stores near schools from 2012 to 2019, with a pronounced rise in those selling ultra-processed products. The results signal the escalating exposure of students to unhealthy options.

Also in a school food environment setting, another study in this Special Issue (**Silva et al.—contribution 3**) compared contexts across different cities and found strong associations between the school food environments and adolescent food consumption, but only in the larger municipalities. Those findings revealed the impact of urban health and underscored how rapid urban growth impacts exposure to unhealthy food environments. Together, these studies advance an urban health lens on school food environments in LMICs.

Extending the organizational domain perspective, though firmly remaining within it, from food environments in schools to those at universities, **Perez et al. (contribution 4)** assessed how the pre- and post-pandemic periods affected a university food environment. Their findings include a decline in vendor numbers, reduced availability of fresh food, increased ultra-processed offerings, and price hikes, emphasizing the vulnerability of institutional food environments during crises and the need for resilient procurement and governance arrangements.

A cross-sectional household study **(Castro Junior et al., contribution 5)** explores how the perceived dimensions of a food environment (such as food availability, quality, and price) are linked to food insecurity. Ultra-processed foods are perceived as cheaper and more diverse than fruits and vegetables, regardless of the actual level of foods’ safety. Households reporting poor perceptions of fruit and vegetable availability and prices faced a significantly higher risk of moderate or severe food insecurity.

At the neighborhood scale, inequalities between local food environments could lead to vulnerable populations experiencing difficulties accessing healthy options. **Craveiro et al. (contribution 6)** analyze consumer food environments, finding that socially highly vulnerable areas suffer from lower variety and quality of fruits and vegetables, and that supermarkets in such areas are less likely to offer a broad variety of vegetable options compared to areas that are less socially vulnerable.

**Structural Inequities, Favelas, and Locally Tuned Models**

In this context pertaining to inequalities in LMICs, **Rocha et al. (contribution 7)** aimed to develop a conceptual model of the relationship between access to food and the favela food environment and its determinants. Inequalities are amplified in favelas, affecting access to basic sanitation, health, education services, and food. This conceptual study models food access within favelas by delineating multiple interacting domains: the individual scale, the micro-environment, the macro-environment, and decision-making. It aims to guide research and policy in spatially vulnerable settings, and underscores that locally nuanced models are essential for effective interventions.

Finally, the approach adopted by **Zeitler et al. (contribution 8)** focuses on Indigenous and rural food environments undergoing transition in LMICs, and they develop a simple and rapid tool for collecting perceptions on and preferences regarding different types and characteristics of food environments, effectively introducing a rapid decision matrix tool that enables assessment of preferences across informal, formal, wild, and cultivated food environments. The results of the decision matrix can be triangulated using a mixed methodology of geolocated participant observation, participatory mapping, market price comparisons, and qualitative interviews. Despite an ongoing food environment transition, participants preferred natural food environments and emphasized flavor and food safety.

**What the Special Issue Teaches Us**

The studies featured in this Special Issue provide critical insights into the diverse contexts and structural challenges that shape food environments in LMICs. Collectively, they underscore the dual importance of both perceived and physical access to healthy foods, while also highlighting the pervasive availability of ultra-processed products. The findings illustrate how pandemics and other public health emergencies disrupt food availability and affordability, and how social vulnerability profoundly influences food access. Overall, the Special Issue demonstrates the urgent need for frameworks and instruments tailored to the realities of LMICs, which will take into account informal economies, rapid urbanization, contextual diversity, regulatory gaps, and deep-seated structural inequities.

This thematic arc aligns with and extends regional scholarship: Gálvez Espinoza et al. (2017) [3], working in Latin America, particularly in Chile, highlighted structural inequities, food sovereignty, and the influence of globalized food markets, all of which are critical for understanding food access and health disparities in marginalized LMIC populations. Downs et al. (2020) [4] proposed measurable indicators tailored to informal settings, while Gupta et al. (2023) [5] emphasized the roles played by power dynamics and corporate influence in shaping LMIC food systems. Popkin and Reardon (2018) [6], in their review, document in depth the recent history of the rapid growth and transformation of the broad food system in the Latin America and the Caribbean (LAC) region, including examination of the rapid rise of supermarkets, large food processing concerns, fast food chains, and food logistics firms. The current state of the art across the region points to the rising availability of ultra-processed foods, shifts in food retail dynamics, and the coexistence of undernutrition and overweight within the same communities.

**Priorities for Research and Action**

Several promising directions take shape: there is a growing push to develop and validate instruments that enable the collection of primary data that will reflect the complexity of food environments in LMICs; more work is being undertaken to understand the policy levers that can reshape food environments; and future studies should consider longitudinal designs (that can track how food environments change over time and how they causally affect diet and nutrition), mixed-methods approaches, natural experiments, and interventions grounded in local realities. Further priorities include greater attention to informal food retail, as well as rural and peri-urban areas whose unique dynamics remain underexplored. Research on how extreme weather events and climate variability affect food environments in LMICs is also urgently needed. Finally, other critical gaps include evaluating multiple dimensions of the food environment, examining interconnections between different settings (including digital and physical environments), and incorporating sustainability considerations.

Policymakers must support national initiatives to monitor food environments, such as those already underway in Mexico, and strengthen collaborations between researchers, communities, and governments. Such efforts must prioritize equity, community voice, and accountability to ensure that research translates into meaningful, context-sensitive action.

**A Call to Deepen, Broaden, and Include**

To truly understand and transform food environments in LMICs, research must be grounded in context, integrating quantitative and qualitative evidence with policy perspectives, and it must also remain vigorously interdisciplinary. Advancing this field requires the production of policy-relevant science that addresses the complex realities of LMICs. Therefore, we urge researchers, policymakers, and practitioners to utilize scientific evidence to promote healthier and more equitable food environments, particularly in vulnerable settings. Changing diets requires changing environments and food systems towards health and sustainability; changing environments, in turn, requires science that is deeper, broader, and more inclusive.
